# Gastric Ulcer From Prolonged Oral Iron Therapy: A Case Report and Literature Review

**DOI:** 10.7759/cureus.67905

**Published:** 2024-08-27

**Authors:** Sandesh R Parajuli, Fnu Vikash, Shefali Amin, Manish Shrestha, Anish Paudel, Anthony Donato

**Affiliations:** 1 Internal Medicine, Reading Hospital, Tower Health, Reading, USA; 2 Gastroenterology and Hepatology, Montefiore Medical Center, Albert Einstein College of Medicine, Bronx, USA; 3 Internal Medicine, Jacobi Medical Center, Albert Einstein College of Medicine, Bronx, USA; 4 Internal Medicine, Tower Health Medical Group, Reading, USA

**Keywords:** oral iron therapy, iron therapy, iron-deficiency anemia, epigastric pain, endoscopy, anemia

## Abstract

Iron deficiency is a leading cause of anemia worldwide and is commonly treated with oral iron supplements, which are known for their GI side effects. We present a case of a 66-year-old woman with a history of GI bleeding and multiple comorbidities who developed a gastric ulcer after prolonged oral iron therapy. Although GI side effects are frequent with iron supplements, ulceration is rare, with only a few documented cases. Endoscopic and histopathological evaluations identified iron deposition in the ulcer bed, confirming the diagnosis. Discontinuing the oral iron led to the resolution of symptoms. This case underscores the importance of recognizing and managing iron-induced gastric ulcers to ensure safe and effective treatment of iron deficiency.

## Introduction

Anemia affects nearly one-fourth of the global population, with iron deficiency being the most common cause [1]. Treatment for iron deficiency often starts with oral iron supplements, which are recommended after identifying sources of blood loss [2]. Depending on the clinical situation, different forms of iron delivery may be used, including food fortification, oral supplements, or intravenous therapy [3]. Oral iron, typically administered in pill or tablet form, is widely used but can cause GI side effects such as nausea, abdominal pain, diarrhea, bloating, constipation, and black-colored stools. Gastric mucosal ulceration is a rare but serious side effect that gastroenterologists should be aware of. Patients with such ulcers should be switched to alternative forms of iron supplementation, and iron tablets should be avoided in those with existing gastric ulcers [3,4]. This case report describes a 66-year-old woman who developed a gastric ulcer as a result of oral iron tablets.

## Case presentation

A 66-year-old morbidly obese female with a history of GI bleeding due to small bowel arteriovenous malformations (AVMs) presented to the hospital with a new melanotic stool. Her medical history included coronary artery disease, heart failure, aortic stenosis, hypertension, hyperlipidemia, and chronic kidney disease stage II. She had recently been prescribed apixaban 5 mg twice daily for paroxysmal atrial fibrillation. Her other home medications included metformin, amiodarone, metoprolol succinate, and pantoprazole 40 mg once daily. She had also been taking ferrous sulfate (325 mg) once daily for over six months. She denied using NSAIDs, bismuth subsalicylate, or additional aspirin or antiplatelet agents. On examination, she was hemodynamically stable, and her blood work, as shown in Table 1, indicated that her hemoglobin and creatinine levels were at baseline.

**Table 1:** Laboratory values

Lab tests	Result	Reference range
Hemoglobin	9.9	12-16 g/dl
Mean corpuscular volume	97	80-99 fL
Ferritin	150	11-307 ng/mL
Iron saturation	32	11-50%
Total iron-binding capacity	299	284-507 ug/dL
Blood urea nitrogen	19	9-23 mg/dL
Creatinine	0.75	0.55-1.02 mg/dL

**Figure 1:**
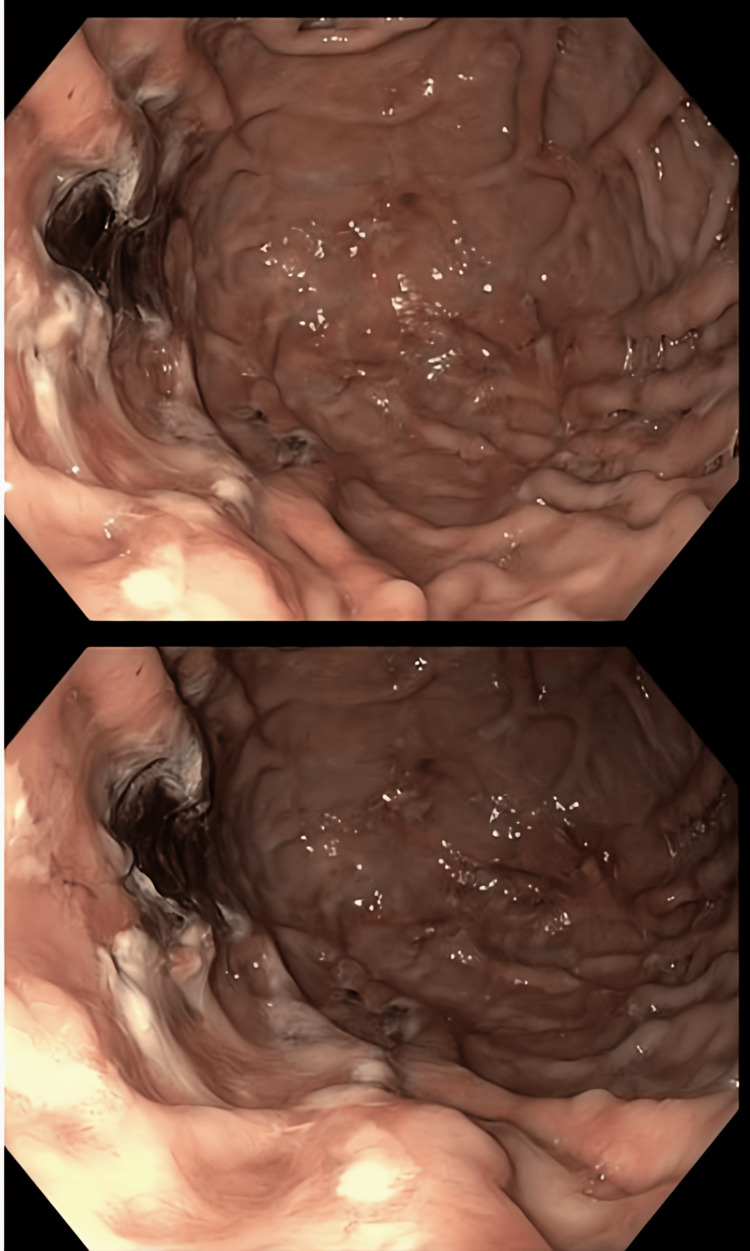
Esophagogastroduodenoscopy image showing the gastric ulcer situated along the proximal gastric body

A CT scan of her abdomen and pelvis with contrast performed in the emergency department did not reveal any obvious source of contrast extravasation. Considering her recent initiation of apixaban, history of AVMs, and low hemoglobin, gastroenterology was consulted for potential endoscopic evaluation. During the upper GI endoscopy, a large (1.5 cm) friable ulcerated mucosa with central eschar was identified along the proximal gastric body (Figure 1).

**Figure 2:**
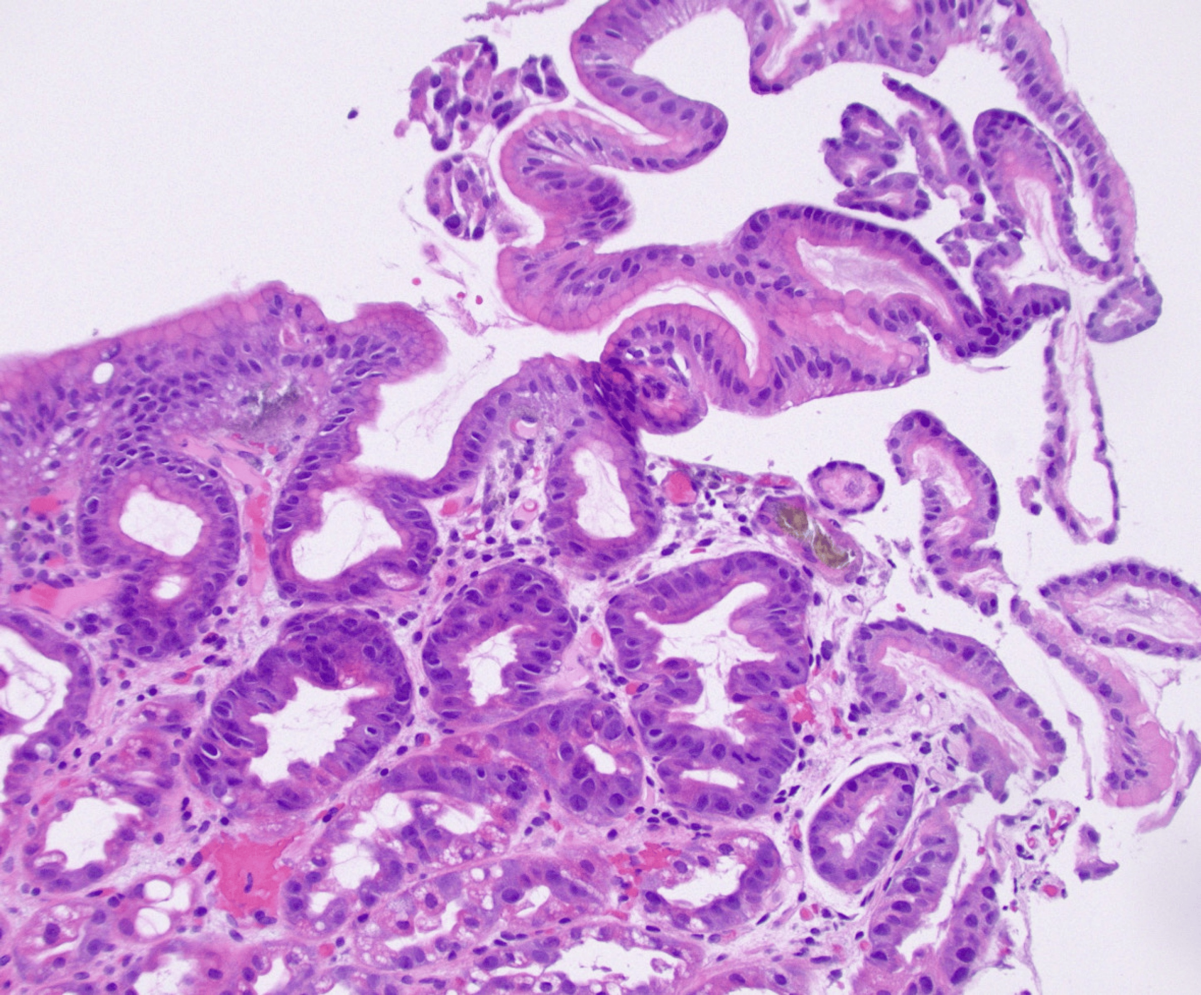
Microscopic slide displaying brownish to golden crystalline iron deposits on the ulcer bed of the eroded gastric mucosa

The upper GI endoscopy otherwise appeared normal, with no visible bleeding. A biopsy was performed, showing an eroded epithelial lining with an infiltrated and edematous lamina propria. The presence of brownish to golden crystalline material within the ulcer bed suggested iron deposition, which was further confirmed using Perl’s stain (Figure 2).

The biopsy results were negative for *Helicobacter pylori *and malignancy. The patient was advised to take pantoprazole 40 mg twice daily until follow-up and to resume oral apixaban. She was instructed to discontinue oral iron supplements, and a follow-up upper GI endoscopy was recommended in two to three months to assess for healing.

## Discussion

Iron-induced gastric ulcers are a notable adverse effect of oral iron supplements used to treat iron deficiency anemia. The GI side effects of these supplements can range from mild discomfort to severe gastric ulceration. The mucosal injury occurs through both direct and indirect mechanisms [4]. Directly, iron supplements can damage the mucosa as they dissolve in gastric acid, releasing free iron ions that catalyze the formation of reactive oxygen species. This disrupts mucosal integrity and contributes to ulcer formation. Indirectly, the increased acidity from oral iron pills can alter the gut microbiota, exacerbating mucosal inflammation. Notably, liquid iron preparations have not been associated with such mucosal ulceration and represent a promising alternative [5-7].

Clinically, iron-induced gastric ulcers present as epigastric pain, often described as gnawing or burning, which may worsen with food intake or improve temporarily with antacids [8]. Nausea, potentially accompanied by vomiting, and, in severe cases, hemoptysis from active bleeding at the ulcer site are also common symptoms [9].

Diagnosis involves a comprehensive approach including patient history, endoscopy, and histopathological analysis. Endoscopy is crucial for visualizing ulcers and differentiating them from other causes, such as *H. pylori *infection or malignancy [6]. Although endoscopic and radiological findings might suggest malignancy, biopsy and histological analysis are necessary for an accurate diagnosis. A key diagnostic clue is the resolution of ulcers following the cessation of oral iron [10].

Management focuses on alleviating symptoms, promoting mucosal healing, and preventing recurrence while meeting the patient’s iron needs [9]. Discontinuation of oral iron, which often significantly reduces symptoms and initiates mucosal healing, is the primary step. Proton pump inhibitors (PPIs) are the preferred pharmacologic treatment, as they accelerate healing and alleviate symptoms in cases of iron-induced gastric damage [9]. The development of ulcers despite PPI use suggests that existing ulcers were exacerbated by oral iron, highlighting the importance of avoiding oral iron supplements in patients with gastric ulcers. H2 receptor antagonists, such as ranitidine, may be used but are generally less effective than PPIs [11]. Sucralfate, which forms a protective barrier over gastric ulcers, can also aid in healing and symptom relief. For patients requiring ongoing iron supplementation, alternative routes like intravenous iron should be considered to prevent further gastric irritation [6]. Additionally, patients should avoid alcohol and smoking, as these can impair mucosal healing [12]. Preventive strategies include using ferrous sulfate with food, prescribing enteric-coated or delayed-release formulations, and appropriate dosing to prevent excessive iron accumulation [13,14].

## Conclusions

Iron-induced gastric ulcers are an underrecognized risk of oral iron therapy, particularly in patients with refractory iron deficiency anemia. Despite the use of PPIs, the risk of mucosal damage persists. Early endoscopic evaluation is crucial for diagnosing these ulcers. Switching to liquid or intravenous iron supplementation can help prevent further mucosal injury and offer a safer management alternative. Early recognition of this complication and timely adjustment of treatment are essential to avoid the severe consequences of iron-induced gastric ulcers.
